# Burnout syndrome and engagement among critical care providers: a cross-sectional study

**DOI:** 10.5935/0103-507X.20200066

**Published:** 2020

**Authors:** Carolina Sant’Anna Antunes Azevedo Castro, Karina Tavares Timenetsky, Marcelo Katz, Thiago Domingos Corrêa, Andre Carvalho Felício, Tais Moriyama, Ana Merzel Kernkraut, Leonardo José Rolim Ferraz, Ary Serpa Neto

**Affiliations:** 1 Department of Critical Care Medicine, Hospital Israelita Albert Einstein - São Paulo (SP), Brazil.; 2 Department of Cardiology, Hospital Israelita Albert Einstein - São Paulo (SP), Brazil.; 3 Brain Institute, Hospital Israelita Albert Einstein - São Paulo (SP), Brazil.; 4 Department of Mental Health, Instituto Bairral - São Paulo (SP), Brazil.; 5 Department of Psychology, Hospital Israelita Albert Einstein - São Paulo (SP), Brazil.

**Keywords:** Step-down unit, Burnout, psychological, Work engagement, Depression, Acute stress disorders, Intensive care units, Unidade semi-intensiva, Esgotamento psicológico, Engajamento no trabalho, Depressão, Transtornos do estresse agudo, Unidades de terapia intensiva

## Abstract

**Objective:**

To evaluate the frequency of severe burnout syndrome among critical care providers and to correlate it with work engagement.

**Methods:**

A self-administered survey including the Maslach Burnout Inventory, Depression Anxiety and Stress Scales, and Gallup questionnaire was distributed. All analyses were stratified by setting (intensive care unit or step-down unit) and by professional group (nurses versus physicians *versus* physiotherapists).

**Results:**

Between February 2017 and June 2017, 206 out of 325 invited professionals (63.4%) answered the questionnaires. Of these, 55 were physicians (26.7%), 88 were physiotherapists (42.7%) and 63 were nurses (30.6%). The frequency of severe burnout was 34.3% (27.9 - 41.4%), and no difference was found between professional groups or settings. The frequency of severe or very severe cases of depression, anxiety or stress was 12.9%, 11.4% and 10.5%, respectively. The median (interquartile range) score observed on the Gallup questionnaire was 41 (34 - 48), and no differences were found between professional groups or settings. There was a negative correlation between burnout and work engagement (r = -0.148; p = 0.035).

**Conclusion:**

There is a high frequency of severe burnout among critical care providers working in the intensive care unit and step-down unit. There was a negative correlation between burnout and work engagement.

## Abstract (pt)

**Objetivo:**

Avaliar a frequência de síndrome de *burnout* grave em profissionais de terapia intensiva e correlacioná-la com o engajamento com o trabalho.

**Métodos:**

Foi distribuído um questionário autoaplicável que incluía o Inventário de *Burnout* de Maslach, a Escala de Depressão Ansiedade e Estresse e o questionário Gallup. Todas as análises foram estratificadas por local de trabalho (unidade de terapia intensiva ou unidade semi-intensiva) e por grupo profissional (enfermeiros *versus* médicos *versus* fisioterapeutas).

**Resultados:**

Entre fevereiro de 2017 e junho de 2017, 206 dos 325 profissionais convidados (63,4%) responderam aos questionários. Destes, 55 eram médicos (26,7%), 88 eram fisioterapeutas (42,7%) e 63 eram enfermeiros (30,6%). A frequência de burnout grave foi de 34,3% (27,9 - 41,4%), e não se identificaram diferenças entre os grupos profissionais ou locais de trabalho. A frequência de casos graves ou muito graves de depressão, ansiedade ou estresse foi de 12,9%, 11,4% e 10,5%, respectivamente. O escore mediano (intervalo interquartil) observado pelo questionário Gallup foi 41 (34 - 48), e não se observaram diferenças entre os grupos profissionais ou locais de trabalho. Houve correlação negativa entre burnout e engajamento com o trabalho (r = -0,148; p = 0,035).

**Conclusão:**

A frequência de burnout grave foi elevada entre os profissionais de saúde que trabalham na unidade de terapia intensiva e na unidade semi-intensiva. Existe uma correlação negativa entre burnout e engajamento com o trabalho.

## INTRODUCTION

Burnout syndrome is defined as a condition of work-related psychological distress associated with physiological changes due to stress.^(1)^ It is characterized by physical, psychological and emotional exhaustion due to excessive effort exerted at work.^(1,2)^ Burnout syndrome includes three interdependent multidimensional factors: emotional exhaustion, depersonalization, and reduced personal accomplishment.^(1,2)^

Professionals usually experience burnout syndrome mainly when the nature of their work demands direct contact with other human beings, such as in the healthcare area.^(3,4)^ Among all hospital settings, the intensive care unit (ICU) has been highlighted as one of the most stressful places, not only among the patients and their relatives but also among the healthcare providers.^(5-9)^ Data on burnout syndrome among professionals working exclusively in step-down units (SDU) are still scarce.

The presence of burnout syndrome in healthcare professionals can impair the quality of care provided to the patient and worsen the quality of life for providers.^(10)^ Additionally, the syndrome is associated with deleterious consequences, including low work engagement, absenteeism, increased rates of job turnover, low patient satisfaction, and decreased quality of service.^(5,9,10)^ Nevertheless, the impact of burnout syndrome on work engagement among critical care providers has not been assessed so far.

Thus, the aim of the present study was to evaluate the frequency of severe burnout syndrome among critical care providers working in an ICU or SDU and to correlate it with work engagement. We hypothesized that the frequency of burnout syndrome is high and is negatively correlated with work engagement.

## METHODS

A survey was carried out using a self-administered questionnaire.

The study was approved by the Institutional Review Board of the *Hospital Israelita Albert Einstein* (CAAE: 58687216.0.0000.0071). All participants signed an online consent form, as approved by the Institutional Review Board.

This study was conducted in the ICU and SDU of a tertiary private 646-bed teaching hospital in São Paulo, Brazil. The ICU comprised 40 beds; the SDU comprised 95 beds and received case-mix patients. There was active assistance by professionals 24 hours a day, 7 days a week. Both the ICU and the SDU had a 24-hour visitation policy (free access at all times; visitors may take turns visiting patients and stay overnight with patients).

During the research, there were 325 healthcare professionals (64 physicians, 155 nurses, and 106 physiotherapists) working in the hospital ICU and SDU. The professional-to-bed ratio in the ICU was 1:10 for physicians (day and night) and 1:5 for nurses (day and night); the ratio was 1:5 for physiotherapists during the daytime and 1:10 during the night. In the SDU, the professional-to-bed ratio was 1:20 for physicians during the daytime and 1:40 during the night, 1:7 for nurses during the daytime and 1:10 during the night, and 1:6 for physiotherapists during the daytime and 1:20 during the night. Healthcare professionals were independent (i.e., they work only in the ICU or SDU), and the working hours (duration and number of shifts) were the same in both units.

All critical care providers (physicians, nurses and physiotherapists) invited to participate in the study were sent an invitation email. Participation was voluntary, and responses remained anonymous.

A self-administered survey in Research Electronic Data Capture (REDCap) was used for data collection. Demographic data were collected, and three instruments were included: the Maslach Burnout Inventory (MBI), to assess burnout syndrome;^(11,12)^ the Depression Anxiety and Stress Scale (DASS-21);^(13-15)^ and the Gallup questionnaire (Supplementary material).^(16)^ All instruments were translated into and validated in the Portuguese language.

The MBI is a 22-item questionnaire that assesses the three domains of burnout: 1) emotional exhaustion (nine items); 2) depersonalization (five items); and 3) personal accomplishment (eight items).^(11,12)^ The DASS-21 is a 21-item instrument used to assess depression (seven items), anxiety (seven items), and stress (seven items).^(13-15)^ Finally, the Gallup questionnaire is a 12-question questionnaire used to assess work engagement using a Likert scale from 1 (‘strongly disagree’) to 5 (‘totally agree’) (Supplementary material).^(16)^

For the diagnosis of severe burnout syndrome, well-established cut-offs were defined for each dimension of the MBI. A score of ≥ 27 points indicated high levels of emotional exhaustion; a score of ≥ 10 points indicated depersonalization, and a score of ≤ 33 points indicated low levels of personal accomplishment.^(11)^ Severe burnout was diagnosed when high levels of emotional exhaustion, depersonalization and low levels of personal accomplishment were found.

For the DASS-21 instrument responses, a score of ≥ 21 points was used to indicate severe depression, ≥ 15 points was used to indicate severe anxiety, and ≥26 points was used to indicate severe stress.^(13)^ Finally, there was no validated cut-off for the Gallup questionnaire. Therefore, in the present study, the responses were considered on a continuous scale ranging from 12 to 60, with higher levels representing higher engagement.

### Statistical analysis

Data are presented as the median (interquartile range) and absolute and relative frequencies. The frequency of severe burnout is shown as a percentage and 95% confidence interval (95%CI). The results from the MBI and DASS-21 are presented according to well-established and defined cut-offs and as the total score according to the sum of responses, ranging from 0 to 132 for the MBI and from 0 to 63 for the DASS-21, with higher levels indicating more burnout (MBI), depression, anxiety or stress (DASS-21). The Gallup questionnaire was assessed as a total score, as described above.

All analyses were stratified according to the setting (ICU *versus* SDU) and professional group (nurses *versus* physicians *versus* physiotherapists). A comparison among groups was made using the Mann-Whitney, Kruskal-Wallis and/or Fisher’s exact test as appropriate. To compare the degrees of burnout, depression, anxiety and stress among the settings and professional groups, a Cochran-Armitage test was used to take into account the ordinal scale of the instruments.

To assess the factors associated with severe burnout syndrome, two approaches were used. First, critical care providers coded as having severe burnout were compared to critical care providers without burnout. Then, the variables with significant differences between these two groups (considering a p < 0.05) were included in a mixed-effect model considering the setting and professional group as random-effects. Variables with a p < 0.05 in this final model were considered independent factors associated with burnout. Finally, the correlations between the MBI, DASS-21 and Gallup scores were assessed through scatterplots, Pearson’s correlation and linear models.

As additional analyses, the frequency of severe burnout was assessed in critical care providers with and without positive screening for depression according to the DASS-21, with providers classified as without depression when the DASS-21 score was < 10. Additionally, the frequency of severe burnout was assessed in critical care providers working exclusively in the hospital. Due to the multiple comparisons, the p value for these additional analyses was corrected using the Bonferroni correction; the p value was significant when < 0.005.

The significance level was set at 0.05 (when not described otherwise), and all analyses were conducted with R v.3.4.0 (R Foundation for Statistical Computing, Vienna, Austria).

## RESULTS

Between February 2017 and June 2017, 206 professionals out of the 325 invited (62.4%) answered the survey. Of these, 55 were physicians (26.7%), 88 were physiotherapists (42.7%) and 63 were nurses (30.6%). Most of the professionals who answered the survey were from the ICU (57.8% [119/206]). The highest rate of response was among physicians (85.9%), followed by physiotherapists (83.0%) and then nurses (40.6%).

The baseline characteristics of the participants are shown in table 1. The median (IQR) age of the participants was 35 (31 - 39) years. The majority of them were female, married, and postgraduates. The majority of participants worked between 3 and 5 days at the hospital and did not work at another hospital. All characteristics were similar between settings (Table 1).

**Table 1:** Characteristics of the included participants

Demographic characteristics	Overall (n = 206)	ICU (n = 119)	SDU (n = 87)	p value
Age (years)	35 (31 - 39)	36 (31 - 4)	34 (30 - 38)	0.053
Male sex	56/206 (27.2)	34/119 (28.6)	23/87 (26.2)	0.708
Marital status				
Single	69/206 (33.5)	44/119 (37.0)	26/87 (29.8)	
Married	108/206 (52.4)	60/119 (50.4)	48/87 (54.8)	0.579
Divorced	10/206 (4.8)	5/119 (4.2)	3/87 (3.6)	
STable union	19/206 (9.3)	9/119 (7.6)	10/87 (11.9)	
Degree				
Graduate	17/206 (8.2)	12/119 (10.1)	2/87 (2.4)	
Specialization	142/206 (68.9)	75/119 (63.0)	69/87 (79.8)	0.038
Masters	23/206 (11.2)	15/119 (12.6)	8/87 (9.5)	
Doctorate	15/206 (7.3)	9/119 (7.6)	6/87 (7.1)	
Postdoctorate	9/206 (4.4)	8/119 (6.7)	1/87 (1.2)	
Religion				
Atheism	9/206 (4.4)	5/119 (4.2)	4/87 (4.8)	
Agnosticism	10/206 (4.9)	6/119 (5.0)	4/87 (4.8)	
Spiritism	42/206 (20.4)	24/119 (20.2)	19/87 (21.4)	0.975
Judaism	3/206 (1.5)	1/119 (0.8)	2/87 (2.4)	
Buddhism	3/206 (1.5)	2/119 (1.7)	1/87 (1.2)	
Christianity	134/206 (65.0)	77/119 (64.7)	56/87 (64.3)	
Other	4/206 (1.9)	2/119 (1.7)	2/87 (6.0)	
Comorbidities				
Hypertension	13/206 (6.3)	11/119 (9.2)	2/87 (2.4)	0.077
Diabetes mellitus	2/206 (1.0)	2/119 (1.7)	0/87 (0.0)	0.512
Heart failure	0/206 (0.0)	0/119 (0.0)	0/87 (0.0)	---
Coronary artery disease	0/206 (0.0)	0/119 (0.0)	0/87 (0.0)	---
Rheumatologic disease	2/206 (1.0)	1/119 (0.8)	1/87 (1.2)	0.999
Insomnia	14/206 (6.8)	10/119 (8.4)	4/87 (4.8)	0.404
COPD	0/206 (0.0)	0/119 (0.0)	0/87 (0.0)	---
Cancer	0/206 (0.0)	0/119 (0.0)	0/87 (0.0)	---
Asthma	4/206 (2.0)	0/119 (0.0)	4/87 (4.8)	0.055
Depression	5/206 (2.4)	3/119 (2.5)	2/87 (2.4)	0.682
Hypothyroidism	7/206 (3.4)	3/119 (2.5)	4/87 (4.8)	0.613
Other	37/206 (18.0)	25/119 (21.0)	12/87 (13.8)	0.221
Pain	106/206 (51.5)	62/119 (52.1)	44/87 (50.5)	0.981
Daily	31/106 (29.1)	23/62 (37.1)	8/44 (18.2)	
3 times a week	37/106 (34.9)	22/62 (35.5)	15/44 (34.1)	0.072
Once a week	27/106 (25.5)	14/62 (22.6)	13/44 (29.5)	
Rarely	10/106 (9.4)	3/62 (4.8)	7/44 (15.9)	
Daily tasks				
Take care of home	149/206 (72.3)	82/119 (68.9)	67/87 (77.0)	0.084
Take care of relatives	52/206 (25.2)	33/119 (27.7)	19/87 (21.8)	0.411
Knowledge about burnout syndrome				0.141
None	4/206 (1.9)	1/119 (0.8)	3/87 (3.4)	
Some	119/206 (57.8)	66/119 (55.5)	53/87 (60.9)	
A lot	80/206 (38.8)	52/119 (43.7)	28/87 (32.2)	
Professional characteristics				
Professional group				< 0.001
Physiotherapy	88/206 (42.7)	38/119 (31.9)	50/87 (57.4)	
Nurse	63/206 (30.6)	41/119 (34.5)	22/87 (25.3)	
Physician	55/206 (26.7)	40/119 (33.6)	15/87 (17.2)	
Period of time working in the profession (years)	11 (7 - 15)	12 (8 - 16)	10 (7 - 15)	0.165
Period of time working in the hospital (years)	7 (4 - 11)	6 (4 - 10)	7 (3 - 12)	0.761
Days per week working in the hospital				0.646
≤ 2 days	14/206 (6.8)	10/119 (8.4)	4/87 (4.8)	
3 - 5 days	124/206 (60.2)	71/119 (59.7)	53/87 (60.9)	
> 5 days	65/206 (31.6)	38/119 (31.9)	27/87 (31.0)	
Days per week working in another hospital				
None	119/206 (57.8)	69/119 (58.0)	50/87 (57.4)	0.020
≤ 2 days	34/206 (16.5)	27/119 (22.7)	7/87 (8.0)	
3 - 5 days	40/206 (19.4)	18/119 (15.1)	22/87 (25.3)	
> 5 days	10/206 (4.9)	5/119 (4.2)	5/87 (5.7)	

ICU - intensive care unit; SDU - step-down unit; COPD - chronic obstructive pulmonary disease. Data are n/total (percentage) or median (interquartile range).

### Frequency of severe burnout syndrome

**Figure 1:**
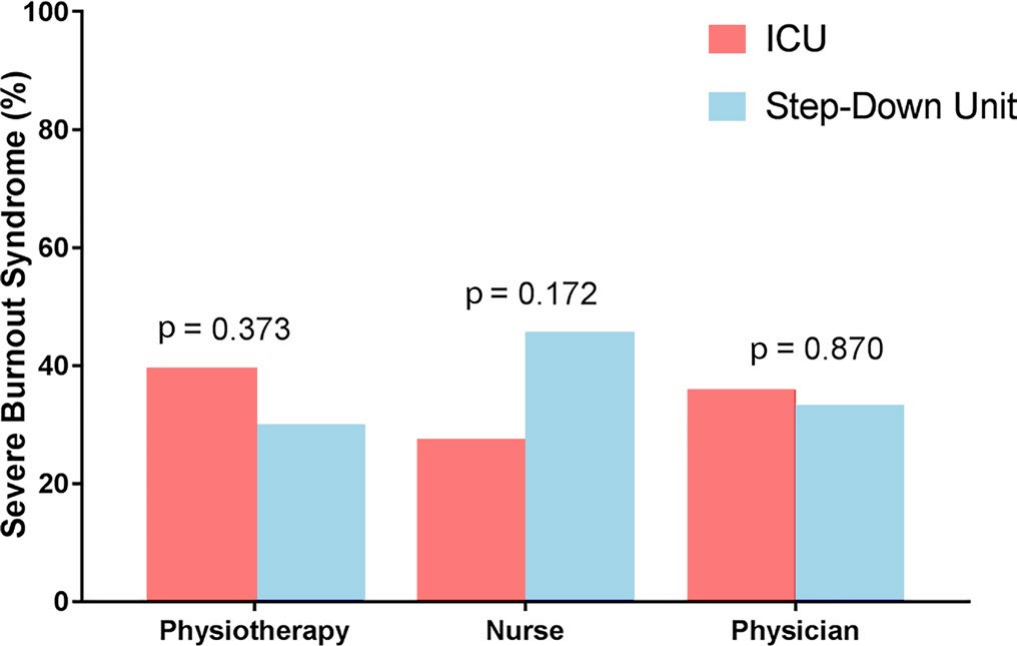
Frequency of severe burnout syndrome according to professional group and setting. ICU - intensive care unit.

The frequency of severe burnout syndrome in the overall cohort was 34.3% (27.9% - 41.4%) without any difference according to the setting (34.2% [25.8% - 43.6%] in the ICU *versus* 34.5% [24.7% - 45.8%] in the SDU; p = 0.960) or professional group (34.1% [24.5% - 45.0%] among physiotherapists *versus* 33.9% [22.6% - 47.1%] among nurses *versus* 35.3% [22.8% - 50.0%] among physicians; p = 0.986) (Table 2 and Figure 1).

**Table 2:** Frequency of severe burnout syndrome, depression, anxiety, stress, and work engagement among participants

	Setting				Professional group			
	Overall (n = 206)	ICU (n = 119)	SDU (n = 87)	p value	Physiotherapists (n = 88)	Nurses (n = 63)	Physicians (n = 52)	p value
MBI								
Total	55 (46 - 68)	55 (48 - 70)	53 (44 - 66)	0.150	54 (43 - 67)	57 (46 - 69)	55 (50 - 70)	0.553
Emotional exhaustion								
Low	21/203 (10.3)	9/119 (7.6)	12/84 (14.3)		13/88 (14.8)	5/63 (7.9)	3/52 (5.8)	
Moderate	102/203 (50.2)	60/119 (50.4)	42/84 (50.0)	0.155	44/88 (50.0)	30/63 (47.6)	28/52 (53.8)	0.245
High	80/203 (39.4)	50/119 (42.0)	30/84 (35.7)		31/88 (35.2)	28/63 (44.4)	21/52 (40.4)	
Depersonalization								
Low	8/202 (4.0)	3/118 (2.5)	5/84 (6.0)		5/88 (5.7)	2/63 (3.2)	1/52 (2.0)	
Moderate	58/202 (28.7)	34/118 (28.8)	24/84 (28.6)	0.408	23/88 (26.1)	18/63 (28.6)	17/52 (33.3)	0.957
High	136/202 (67.3)	81/118 (68.6)	55/84 (65.5)		60/88 (68.2)	43/63 (68.3)	33/52 (64.7)	
Professional accomplishment								
Low	190/201 (94.5)	108/117 (92.3)	82/84 (97.6)		85/87 (97.7)	56/62 (90.3)	49/52 (94.2)	
Moderate	8/201 (4.0)	6/117 (5.1)	2/84 (2.4)	0.073	1/87 (1.1)	4/62 (6.5)	3/52 (5.8)	0.171
High	3/201 (1.5)	3/117 (2.6)	0/84 (0.0)		1/87 (1.1)	2/62 (3.2)	0/52 (0.0)	
Severe burnout	69/201 (34.3)	40/117 (34.2)	29/84 (34.5)	0.960	30/88 (34.1)	21/62 (33.9)	18/51 (35.3)	0.986
DASS-21								
Total	11 (6 - 19)	12 (6 - 23)	10 (6 -16)	0.091	10 (6 - 18)	13 (6 - 23)	12 (5 - 17)	0.484
Depression								
Normal	125/202 (61.9)	64/118 (54.2)	61/84 (72.6)		59/88 (67.0)	39/62 (62.9)	27/52 (51.9)	
Mild	32/202 (15.8)	18/118 (15.3)	14/84 (16.7)	0.001	11/88 (12.5)	9/62 (14.5)	12/52 (23.1)	0.533
Moderate	19/202 (9.4)	16/118 (13.6)	3/84 (3.6)		8/88 (9.1)	5/62 (8.1)	6/52 (11.5)	
Severe	18/202 (8.9)	13/118 (11.0)	5/84 (6.0)		8/88 (9.1)	4/62 (6.5)	6/52 (11.5)	
Very severe	8/202 (4.0)	7/118 (5.9)	1/84 (1.2)		2/88 (2.3)	5/62 (8.1)	1/52 (1.9)	
Anxiety								
Normal	152/202 (75.2)	87/119 (73.1)	65/83 (78.3)		69/88 (78.4)	37/62 (59.7)	46/52 (88.5)	
Mild	9/202 (4.5)	4/119 (3.4)	5/83 (6.0)	0.317	3/88 (3.4)	5/62 (8.1)	1/52 (1.9)	0.002
Moderate	18/202 (8.9)	13/119 (10.9)	5/83 (6.0)		4/88 (4.5)	10/62 (16.1)	4/52 (7.7)	
Severe	9/202 (4.5)	6/119 (5.0)	3/83 (3.6)		8/88 (9.1)	1/62 (1.6)	0/52 (0.0)	
Very severe	14/202 (6.9)	9/119 (7.6)	5/83 (6.0)		4/88 (4.5)	9/62 (14.5)	1/52 (1.9)	
Stress								
Normal	124/201 (61.7)	68/117 (58.1)	56/84 (66.7)		54/87 (62.1)	36/62 (58.1)	34/52 (65.4)	
Mild	26/201 (12.9)	13/117 (11.1)	13/84 (15.5)	0.046	12/87 (13.8)	8/62 (12.9)	6/52 (11.5)	0.679
Moderate	30/201 (14.9)	20/117 (17.1)	10/84 (11.9)		12/87 (13.8)	11/62 (7.7)	7/52 (13.5)	
Severe	15/201 (7.5)	11/117 (9.4)	4/84 (4.8)		7/87 (8.0)	4/62 (6.5)	4/52 (7.7)	
Very severe	6/201 (3.0)	5/117 (4.3)	1/84 (1.2)		2/87 (2.3)	3/62 (4.8)	1/52 (1.9)	
Gallup*								
Total	41 (34 - 48)	40 (33 - 47)	43 (34 - 49)	0.239	43 (36 - 49)	40 (31 - 49)	41 (31 - 48)	0.403

ICU - intensive care unit; SDU - step-down unit; MBI - Maslach Burnout Inventory; DASS-21 - Depression, Anxiety and Stress Scale. Results expressed as n/total (percentage) or median (interquartile range).

* Gallup is a 12-question questionnaire used to assess work engagement using a Likert scale from 1 (“strongly disagree”) to 5 (“totally agree”).

The majority of participants showed a moderate level of emotional exhaustion (50.2% [43.2% - 57.3%]), a high level of depersonalization (67.3% [60.3% - 73.6%]), and a low level of personal accomplishment (94.5% [90.2% - 97.1%]). There was no difference in the components of the MBI or the total score among the settings or professional groups (Table 2).

### Assessment of depression, anxiety, stress, and work engagement

The frequency of severe or very severe cases of depression, anxiety and stress was 12.9% (8.7% - 18.5%), 11.4% (7.5% - 16.8%), and 10.4% (6.7% - 15.7%), respectively (Table 2). There was a difference in the frequency of depression and stress according to the setting, with professionals working in the ICU presenting with a higher frequency of severe or very severe cases (p = 0.001 for depression and p = 0.046 for stress) (Table 2). Additionally, a higher frequency of very severe cases of anxiety was found among nurses (p = 0.003).

The median (IQR) score on the Gallup questionnaire was 41 (34 - 48), with no difference regarding setting (p = 0.239) or professional group (p = 0.403) (Table 2 and Table 1S - Supplementary material).

### Factors associated with severe burnout syndrome

The characteristics of critical care providers with and without severe burnout syndrome are shown in table 2S (Supplementary material). There was a difference in marital status (p = 0.036), a higher frequency of pain (p = 0.047) and a higher number of days working in another hospital (p = 0.020) in critical care providers with severe burnout syndrome (Table 2S - Supplementary material). In addition, providers with severe burnout syndrome had higher scores on all subcomponents of the DASS-21 (p = 0.001 for depression and anxiety and p < 0.001 for stress) and lower work engagement according to the Gallup questionnaire (p = 0.014) (Table 2S - Supplementary material).

In the multivariable analysis, moderate-to-severe levels of stress (adjusted odds ratio (OR) 5.54 [95%CI, 1.78 - 18.63]; p = 0.004 for moderate levels, and OR 11.47 [1.68 - 109.34]; p = 0.017 for severe levels) and working between 3 and 5 days in another hospital (OR 3.61 [1.53 - 8.75]; p = 0.003) were independently associated with a higher risk of severe burnout syndrome (Table 3).

**Table 3:** Factors associated with severe burnout syndrome in the multivariable analysis

	Odds ratio (95%CI)	p value
DASS-21 anxiety		
Normal	1 (Reference)	
Mild	1.56 (0.24 - 7.91)	0.658
Moderate	1.02 (0.28 - 3.50)	0.985
Severe	1.14 (0.16 - 10.64)	0.931
Very severe	0.63 (0.09 - 4.06)	0.696
DASS-21 depression		
Normal	1 (Reference)	
Mild	0.52 (0.17 - 1.49)	0.175
Moderate	0.62 (0.15 - 2.39)	0.516
Severe	0.94 (0.18 - 4.95)	0.989
Very severe	0.18 (0.01 - 3.36)	0.211
DASS-21 stress		
Normal	1 (Reference)	
Mild	2.25 (0.77 - 6.57)	0.144
Moderate	5.54 (1.78 - 18.63)	0.004
Severe	11.47 (1.68 - 109.34)	0.017
Very severe	25.03 (0.72 - 124.40)	0.070
Pain	1.74 (0.83 - 2.87)	0.117
Marital status		
Single	1 (Reference)	
Married	1.86 (0.85 - 4.21)	0.112
Divorced	0.00 (0.00 - 3.12)	0.987
STable union	2.21 (0.60 - 8.15)	0.157
Gallup total	0.97 (0.93 - 1.01)	0.166
Days per week working in another hospital		
None	1 (Reference)	
≤ 2 days	0.87 (0.27 - 2.60)	0.727
3 - 5 days	3.61 (1.53 - 8.75)	0.003
> 5 days	0.59 (0.07 - 2.94)	0.531

95%CI - 95% confidence interval; DASS-21 - Depression Anxiety Stress Scale.

### Correlation between Maslach Burnout Inventory, Depression Anxiety and Stress Scale and work engagement

**Figure 2:**
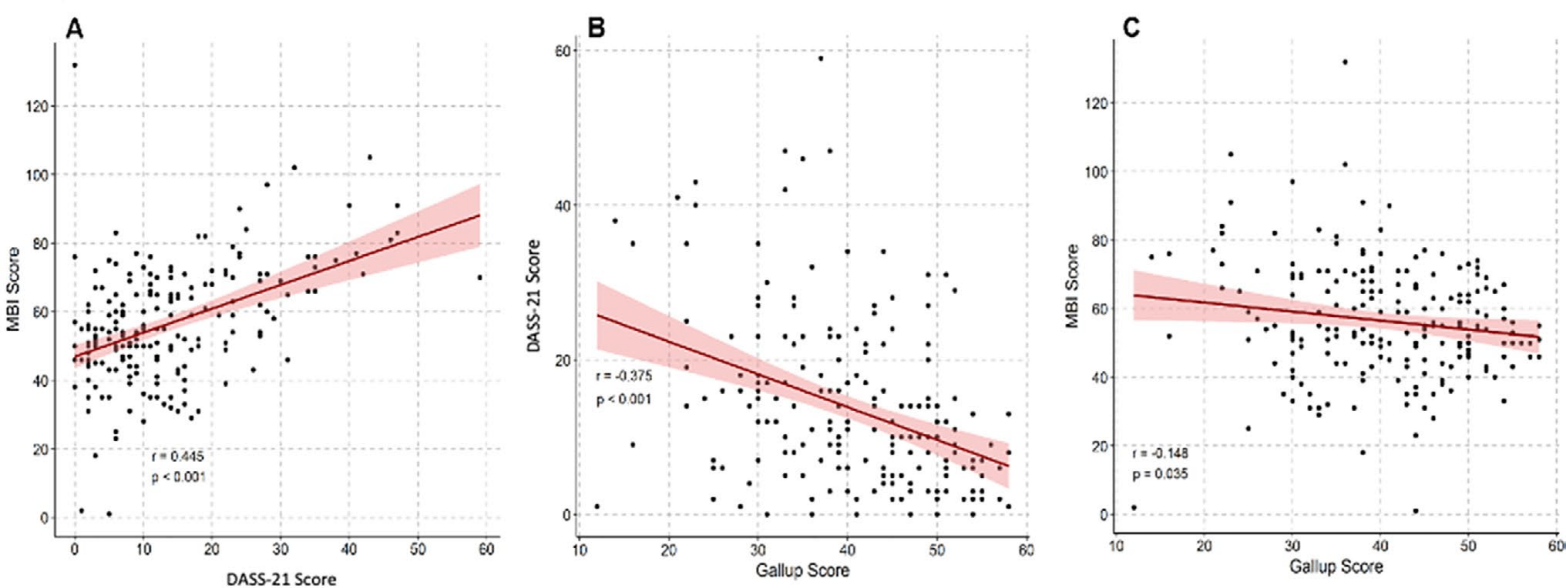
Correlation between (A) Depression Anxiety Stress Scale score and Maslach Burnout Inventory; (B) Gallup and Depression Anxiety Stress Scale; and (C) Gallup and Maslach Burnout Inventory. MBI - Maslach Burnout Inventory; DASS - Depression Anxiety Stress Scale.

There was a positive correlation between the DASS-21 score and the MBI score (r = 0.445; p < 0.001) and a negative correlation between work engagement and the DASS-21 score (r = -0.375; p < 0.001) and between work engagement and the MBI score (r = -0.148; p = 0.035) (Figure 2).

### Additional analyses

After the Bonferroni correction, there was no difference in the frequency of severe burnout syndrome in participants with or without a positive screening for depression (43.4% [32.3% - 55.2%] *versus* 28.8% [21.2% - 37.7%]; p = 0.046) (Table 3S - Supplementary material). Additionally, there was no difference in the frequency of severe burnout syndrome according to the presence or absence of positive screening for depression, setting or professional group (Table 3S - Supplementary materia). Finally, there was no difference in the frequency of severe burnout syndrome between participants working exclusively in the hospital and those who were not (29.7% [21.8% - 38.9%] *versus* 41.0% [30.5% - 52.3%], p = 0.100) (Table 3S - Supplementary material).

## DISCUSSION

In the present study, we showed that there is a high frequency of severe burnout syndrome among critical care providers; there is no difference in this frequency according to setting or professional group; there is an association between both stress and the number of days working in another hospital and risk of severe burnout syndrome; there is a positive correlation between depression, anxiety, stress and burnout; and there is a negative correlation between burnout and work engagement.

The frequency of severe burnout syndrome found in the present study (considering high levels in the three domains that make up the syndrome) was higher than that reported by some previous studies.^(8,17,18)^ The higher frequency of burnout, especially when related to emotional exhaustion and depersonalization, could suggest a high workload and an imbalance between technical skills and interpersonal relationships.^(10,19,20)^ Some studies have shown that individual factors as well as factors related to the organization of the work process increase the predisposition to burnout.^(21)^ Factors associated with an increased burnout risk include high workload, low control over work, low support among coworkers, low recognition and lack of congruence between the ethical and moral values of the worker and those of the institution.^(22)^ Workers with a high level of perfectionism who are very concerned with the outcome of their work are the ones most at risk of burnout.^(23)^

In contrast with other studies, no difference in the frequency of severe burnout syndrome was found according to setting or professional group.^(8,10,20)^ It has been postulated that ICU physicians and nurses have as high a risk of developing severe burnout as other healthcare professionals. Nevertheless, there are limited data available on the frequency of severe burnout in other healthcare providers, such as physiotherapists and respiratory or speech therapists.^(8,10,20)^ One possible explanation for our findings is the daily ICU and SDU clinical rounds involving all critical care providers, allowing them to share decisions and responsibility among all professionals involved in patient care.

Several studies have already reported some risk factors associated with burnout syndrome, such as age, sex, time dedicated to work, professional experience, interpersonal relationships, personality and beliefs, marital status and educational level.^(6,10,24)^ Factors related to work organization have been minimally studied, especially concerning conflicts.^(25)^ Azoulay et al. reported a prevalence of perceived conflicts in up to 70% of ICU staff, and when present, these conflicts were perceived as severe in more than half of the cases and were associated with increased job strain.^(25)^ Moral distress is another important factor, defined as the inability of a moral agent to act according to his or her core values and perceived obligations due to internal and external constraints, which is also considered a conflict. Moral distress is independently associated with the development of burnout syndrome.^(8)^

In the present study, we found a positive association between the period of time spent working in another hospital and a higher risk of burnout. Some studies have shown an association between workload and a higher risk of burnout.^(17,26)^ Health care providers who often work in another hospital during the week (3 to 5 days a week) must deal with different demands of work in each hospital. This may influence the psychological stress suffered as a result of these different job demands. However, those working more than 5 days in another hospital end up having fewer weekdays to deal with different job demands, which probably causes lower psychological stress. Indeed, a higher workload is common in healthcare providers, contributing to burnout and stress. It is important to mention that workload does not depend solely on the number of hours worked but rather on the psychological stress suffered by the demands of work.^(27)^

A higher frequency of musculoskeletal pain among critical care providers was observed in the present study. It has been suggested that despite the psychological stress associated with burnout, the syndrome could also lead to physical problems.^(24)^ Indeed, musculoskeletal disorders appear to be directly associated with burnout syndrome since workers experiencing burnout have a higher risk of short-term and long-term pain compared to professionals not suffering from burnout.^(28,29)^

There was a weak positive correlation between the DASS-21 score and the MBI score. In line with this, some authors suggest a burnout-depression overlap, especially because the instruments used to assess both of them are mainly composed of components related to fatigue.^(2,30-32)^ However, some researchers argue that burnout syndrome and depression are different conditions.^(33,34)^

There is clear evidence that burnout syndrome could also affect the institution the provider works at, since it could lead to a decline in the performance of the professional, causing a direct impact on patient care.^(10)^ For instance, the presence of burnout in nurses has been associated with reduced quality of care, lower patient satisfaction, a higher number of adverse events, higher rates of healthcare-associated infections, and higher 30-day mortality.^(35,36)^

This is the first study assessing the association between burnout syndrome and work engagement among critical care providers. The study was not powered to establish a causal relationship, and there is an ongoing debate about the relationship between engagement and burnout, with discussions over whether they are distinct constructs or whether engagement is just the opposite of burnout.^(37)^ The relationship between burnout and engagement may be due to the worker’s inability to adequately deal with the demands imposed by the work, either for individual reasons or due to the organization of the work process.

The present study presents some limitations. First, it was a single center study, and this should be taken into account when interpreting our results. Second, due to the anonymous and voluntary nature of the interviews, more than 30% of the team and approximately 60% of the nurses did not answer the survey. Three, a small number of questions were not answered, creating some missing data in the analyses. Fourth, individual personalities and conflicts were not assessed. Fifth, there was a risk of selection bias since participation was voluntary, and providers with some degree of burnout could have felt more prone to answer than those without it. Sixth, critical care provider workload was not assessed, which precludes us from determining what its contribution was to the development of severe burnout. Finally, it is important to emphasize that the instruments used in the study are just for screening. For a more detailed diagnosis, a careful evaluation by a psychiatrist is necessary.

## CONCLUSION

In the present study, a high frequency of severe burnout syndrome among critical care providers was found, without differences according to setting or professional group. Additionally, a negative correlation was found between burnout and work engagement.
