# Supplementary material for: Burnout syndrome and engagement among critical care providers: a cross-sectional study
Source: Rev Bras Ter Intensiva. 2020 Jul-Sep;32(3):381–90. doi: 10.5935/0103-507X.20200066 (PMC7595714; doi:10.5935/0103-507X.20200066)
Supplement: Supplementary file 1 [file rbti-32-03-0381-suppl01.pdf]

Burnout syndrome and engagement among critical care providers: a cross-sectional study

Síndrome de burnout e engajamento em profissionais de saúde: um estudo transversal

Carolina Sant'Anna Antunes Azevedo Castro[1], Karina Tavares Timenetsky[1], Marcelo Katz[2], Thiago Domingos Corrêa[1], Andre Carvalho Felício[3], Tais Moriyama[4], Ana Merzel Kernkraut[5], Leonardo José Rolim Ferraz[1], Ary Serpa Neto[1]

GALLUP QUESTIONNAIRE

The Gallup is a 12-question questionnaire used to assess work engagement using a Likert scale from 1 ("strongly disagree") to 5 ("totally agree").(16) The questions are presented below:

1. Do you know what is expected of you at work?
2. Do you have the materials and equipment to do your work right?
3. At work, do you have the opportunity to do what you do best every day?
4. In the last 7 days, have you received recognition or praise for doing good work?
5. Does your supervisor, or someone at work, seem to care about you as a person?
6. Is there someone at work who encourages your development?
7. At work, do your opinions seem to count?
8. Does the mission/purpose of your company make you feel your job is important?
9. Are your associates (fellow employees) committed to doing quality work?
10. Do you have a best friend at work?
11. In the last 6 months, has someone at work talked to you about your progress?
12. In the last year, have you had opportunities to learn and grow?

Table 1S - Gallup questionnaire responses according to setting and professional group

Gallup	Setting			p value	Professional group			p value
	Overall (n = 206)	ICU (n = 119)	SDU (n = 87)		Physiotherapists (n = 88)	Nurses (n = 63)	Physicians (n = 52)	
Total	41 (34 - 48)	40 (33 - 47)	43 (34 - 49)	0.239	43 (36 - 49)	40 (31 - 49)	41 (31 - 48)	0.403
Question 1	4 (3 - 4)	4 (3 - 4)	4 (3 - 5)	0.533	4 (3 - 4)	4 (3 - 5)	4 (3 - 4)	0.880
Question 2	5 (4 - 5)	5 (4 - 5)	5 (4 - 5)	0.063	5 (4 - 5)	4 (4 - 5)	4 (4 - 5)	0.001
Question 3	4 (3 - 5)	4 (3 - 4)	4 (3 - 5)	0.367	4 (4 - 5)	4 (2 - 5)	4 (3 - 5)	0.009
Question 4	2 (1 - 3)	2 (1 - 3)	2 (1 - 4)	0.829	2 (1 - 3)	2 (1 - 4)	2 (1 - 4)	0.679
Question 5	3 (2 - 4)	3 (2 - 4)	3 (2 - 4)	0.961	4 (2 - 4)	3 (2 - 4)	3 (1 - 4)	0.052
Question 6	4 (2 - 4)	3 (2 - 4)	4 (2 - 4)	0.127	4 (3 - 4)	3 (2 - 4)	4 (2 - 4)	0.323
Question 7	3 (2 - 4)	3 (2 - 4)	4 (2 - 4)	0.096	3 (2 - 4)	3 (2 - 4)	3 (2 - 4)	0.122
Question 8	3 (2 - 4)	3 (2 - 4)	4 (3 - 4)	0.105	3 (3 - 4)	3 (2 - 4)	3 (2 - 4)	0.140
Question 9	4 (2 - 4)	3 (2 - 4)	4 (3 - 4)	0.001	4 (3 - 4)	3 (2 - 4)	3 (2 - 4)	0.105
Question 10	4 (2 - 5)	4 (2 - 5)	4 (2 - 4)	0.401	4 (2 - 5)	4 (2 - 5)	3 (2 - 5)	0.938
Question 11	4 (2 - 5)	4 (3 - 5)	4 (2 - 4)	0.342	4 (2 - 4)	4 (2 - 4)	4 (3 - 5)	0.517
Question 12	4 (3 - 5)	4 (3 - 5)	4 (3 - 5)	0.869	4 (2 - 5)	4 (3 - 5)	4 (3 - 5)	0.303

ICU - intensive care unit; SDU - step-down unit. Data expressed as the median (interquartile range).

Table 2S - Characteristics of the critical care providers with and without severe burnout syndrome

	Severe burnout (n = 69)	Without burnout (n = 132)	p value
Demographic characteristics			
Age (years)	35 (31 - 38)	35 (31 - 40)	0.437
Male sex	22/69 (31.9)	34/132 (25.8)	0.357
Marital status			
Single	20/69 (29.0)	49/132 (37.1)	
Married	39/69 (56.5)	66/132 (50.0)	0.036
Divorced	0/69 (0.0)	8/132 (6.1)	
Stable union	10/69 (14.5)	9/132 (6.8)	
Degree			
Graduate	4/69 (5.8)	10/132 (7.6)	
Specialization	46/69 (66.7)	94/132 (7.1)	
Masters	7/69 (10.1)	16/132 (12.1)	0.511
Doctorate	7/69 (10.1)	8/132 (6.1)	
Postdoctorate	5/69 (7.2)	4/132 (3.0)	
Religion			
Atheism	4/69 (5.8)	5/132 (3.8)	
Agnosticism	2/69 (2.9)	8/132 (6.1)	
Spiritism	18/69 (26.1)	24/132 (18.2)	0.679
Judaism	0/69 (0.0)	3/132 (2.3)	
Buddhism	1/69 (1.4)	2/132 (1.5)	
Christianity	39/69 (56.5)	80/132 (60.6)	
Others	5/69 (7.2)	10/132 (7.6)	
Comorbidities			
Hypertension	7/69 (10.1)	6/132 (4.5)	0.139
Diabetes mellitus	1/69 (1.4)	1/132 (0.8)	0.999
Heart failure	0/69 (0.0)	0/132 (0.0)	---
Coronary artery disease	0/69 (0.0)	0/132 (0.0)	---
Rheumatologic disease	0/69 (0.0)	2/132 (1.5)	0.546
Insomnia	7/69 (10.1)	7/132 (5.3)	0.245
COPD	0/69 (0.0)	0/132 (0.0)	---
Cancer	0/69 (0.0)	0/132 (0.0)	---
Others	13/69 (18.8)	24/132 (18.2)	0.908
Pain	42/68 (61.8)	62/132 (47.0)	0.047
Daily	17/42 (40.5)	14/62 (22.6)	
3 times a week	15/42 (35.7)	20/62 (32.2)	0.067
Once a week	9/42 (21.4)	18/62 (29.0)	
Rarely	1/42 (2.4)	9/62 (14.5)	
Daily tasks			
Take care of home	51/69 (73.9)	96/132 (72.7)	0.857
Take care of relatives	15/69 (21.7)	33/132 (25.0)	0.866
Knowledge about burnout syndrome			
None	2/69 (2.9)	2/132 (1.5)	0.499
Feel	37/69 (53.6)	81/132 (61.4)	
A lot	30/69 (43.5)	49/132 (37.1)	

continue...

...continuation

	Severe burnout (n = 69)	Without burnout (n = 132)	p value
Professional characteristics			
Setting			
ICU	40/69 (58.0)	77/132 (58.3)	0.960
Step-down unit	29/69 (42.0)	55/132 (41.7)	
Professional group			
Physiotherapists	30/69 (43.5)	58/132 (43.9)	0.986
Nurses	21/69 (30.4)	41/132 (31.1)	
Physicians	18/69 (26.1)	33/132 (25.0)	
Period of time working in the profession, years	12 (7 - 15)	11 (7 - 16)	0.522
Period of time working in the hospital, years	7 (4 - 10)	7 (3 - 12)	0.989
Days per week working at the hospital			
≤ 2 days	6/69 (8.7)	8/132 (6.1)	0.606
3 - 5 days	39/69 (56.5)	83/132 (62.9)	
> 5 days	24/69 (34.8)	41/132 (31.1)	
Days per week working in another hospital			
None	35/69 (50.7)	83/132 (62.9)	0.020
≤ 2 days	10/69 (14.5)	23/132 (17.4)	
3 - 5 days	22/69 (31.9)	18/132 (13.6)	
> 5 days	2/69 (2.9)	8/132 (6.1)	
Instruments			
MBI total	70 (65 - 73)	50 (41 - 55)	< 0.001
DASS-21 total	15 (9 - 24)	9 (5 - 15)	< 0.001
Depression	4 (2 - 9)	2 (1 - 5)	0.001
Anxiety	2 (1 - 6)	1 (0 - 3)	0.001
Stress	9 (5 - 11)	5 (2 - 8)	< 0.001
Gallup total	39 (31 - 45)	43 (34 - 49)	0.014

COPD - chronic obstructive pulmonary disease; ICU - intensive care unit; MBI - Maslach Burnout Inventory; DASS-21 - Depression Anxiety Stress Scale. Results expressed as n/total (percentage) or median (interquartile range).

Table 3S - Frequency of severe burnout syndrome according to the presence of depression and working exclusively in the hospital

	Setting				Professional class			
	Overall	ICU	SDU	p value*	Physiotherapists	Nurses	Physicians	p value*
Depression								
Positive†	33/76 (43.4)	23/53 (43.4)	10/23 (43.5)	0.999	14/29 (48.3)	9/23 (39.1)	10/24 (41.7)	0.839
Negative	36/125 (28.8)	17/64 (26.6)	19/61 (31.1)	0.693	16/59 (27.1)	12/39 (30.8)	8/27 (29.6)	0.903
p value‡	0.046	0.077	0.313		0.058	0.582	0.396	
Working exclusively in the hospital								
Yes§	35/118 (29.7)	23/68 (33.8)	12/50 (24.0)	0.309	12/49 (24.5)	20/57 (35.1)	3/12 (25.0)	0.450
No	34/83 (41.0)	17/49 (34.7)	17/34 (50.0)	0.180	18/39 (46.1)	1/5 (20.0)	15/39 (38.5)	0.559
p value‡	0.100	0.999	0.019		0.042	0.654	0.501	

ICU - intensive care unit; SDU - step-down unit. Results expressed as n/total (percentage). * Comparison between settings or professional groups among depression categories or working exclusively or not in the hospital ($p < 0.005$ considered significant after Bonferroni correction); † participants classified as screening positive for depression when DASS-21 < 10 (normal category); ‡ comparison between patients with or without positive screening for depression or working exclusively or not in the hospital ($p < 0.005$ considered significant after Bonferroni correction); § working exclusively in the hospital was defined as no other days working in another hospital.
